# Mito‐Nuclear Discordance and Species Boundaries in the Freshwater Fish Genus *Cyprinion* Revealed by Genome‐Wide SNPs

**DOI:** 10.1002/ece3.73893

**Published:** 2026-07-31

**Authors:** Iraj Hashemzadeh Segherloo, Matthias F. Geiger, Eric Normandeau, Jörg Freyhof

**Affiliations:** ^1^ Department of Fisheries Sciences, Faculty of Natural Resources and Earth Sciences Shahr‐e‐Kord University Shahr‐e‐Kord Iran; ^2^ Museum für Naturkunde Leibniz Institute for Evolution and Biodiversity Science Berlin Germany; ^3^ Leibniz Institute for the Analysis of Biodiversity Change, Museum Koenig Bonn NRW Germany; ^4^ Plateforme de Bio‐informatique de l'IBIS (Institut de Biologie Intégrative et des Systèmes) Université Laval Québec Québec Canada

**Keywords:** evolution, genotyping‐by‐sequencing (GBS), hybridization, phylogeography, taxonomy

## Abstract

The cyprinid genus *Cyprinion*, comprising nine species distributed from the Indus drainage across the Iranian Plateau to Mesopotamia and the Arabian Peninsula, exhibits contentious species boundaries due to extensive morphological overlap and limited molecular data. In this study, we integrated mitochondrial DNA (*COI*) sequences and genome‐wide single nucleotide polymorphism (SNP) data to investigate the phylogenetic relationships, taxonomy, and biogeography of six species: *C. acinaces* , *C. kais* , *C. macrostomum* , *C. microphthalmum* , *C. mhalense*, and *C. muscatense*. Mitochondrial analyses revealed extremely shallow divergence among *C. macrostomum* , *C. kais* , *C. acinaces* , and *C. tenuiradius* , sharing identical or closely related haplotypes. In contrast, genome‐wide SNP analyses resolved most taxa as distinct genomic clusters. Clustering and gene‐flow analyses identified five major genetic groups and revealed patterns consistent with historical introgression, particularly between *C. macrostomum* and *C. acinaces* . Despite shallow mitochondrial divergence, genome‐wide SNP analyses revealed differentiation among several taxa, providing preliminary support for lineage divergence. However, these interpretations remain constrained by limited genomic sampling, and alternative explanations including incomplete lineage sorting cannot be excluded. Our results further refine biogeographic hypotheses for *Cyprinion* species, suggesting that recent dispersal and historical connectivity among Mesopotamian, Iranian, and Arabian drainages may have been facilitated by palaeodrainage systems active during the Late Pleistocene. In addition, analysis of unpublished GenBank *COI* sequences suggests that *C. tenuiradius* , previously considered restricted to northern Persian Gulf drainages, may also occur on the Arabian Peninsula. This study provides the first genomic framework for understanding the taxonomy and biogeography of *Cyprinion* in western Asia.

## Graphical abstract

The cyprinid genus Cyprinion exhibits complex and contentious species boundaries across western Asia due to extensive morphological overlap and shallow mitochondrial divergence. Using integrated COI and genome‐wide SNP analyses, we identified distinct genomic clusters among several taxa despite shared mitochondrial haplotypes, revealing signatures of historical introgression and recent divergence while refining biogeographic hypotheses for the group. These findings provide the first genomic framework for understanding the taxonomy, evolutionary history, and regional connectivity of Cyprinion species across Mesopotamian, Iranian, and Arabian drainages.

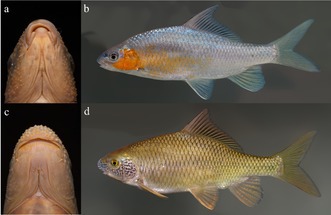

## 1 Introduction

The genus *Cyprinion* comprises morphologically distinctive and ecologically versatile cyprinid fishes distributed from the Indus drainage across the Iranian Plateau to Mesopotamia and the Arabian Peninsula (Freyhof, Yoğurtçuoğlu, et al. 2025). Species of *Cyprinion* inhabit a wide range of aquatic environments, including fast‐flowing mountain streams, perennial wadis, and brackish desert rivers, where they function primarily as algivorous and detritivorous grazers. The genus currently includes nine recognized species: *C. acinaces* , *C. kais* , *C. macrostomum* , *C. mhalense*, *C. milesi* , *C. microphthalmum* , *C. muscatense*, *C. tenuiradius* , and *C. watsoni* (Freyhof et al. 2021; Freyhof, Yoğurtçuoğlu, et al. 2025). Although *Cyprinion* species have no commercial fisheries importance and are not aquaculture or primary recreational fishery targets, they constitute important components of freshwater ecosystems and include several endemic taxa of conservation significance.

Species diversity within *Cyprinion* has long been controversial (Nasri et al. 2013) because morphological differentiation is often subtle and some diagnostic characters may be influenced by ecological adaptation. Mouth morphology, which is closely associated with feeding ecology, has traditionally been regarded as one of the most important taxonomic characters within the genus (Esmaeili et al. 2024; Freyhof, Yoğurtçuoğlu, et al. 2025). However, studies of cyprinid fishes have demonstrated that trophic morphology may exhibit substantial phenotypic plasticity and convergent evolution, potentially reducing its reliability for species delimitation (Nagelkerke and Sibbing 1996, 1998, 2000; Vreven et al. 2016). Consequently, morphological evidence alone may not always provide an adequate basis for resolving species boundaries within *Cyprinion*.

Until recently, molecular information for *Cyprinion* was scarce. Esmaeili et al. (2024) provided the first broad phylogenetic assessment of the genus based on mitochondrial cytochrome oxidase subunit I (*COI*) sequences and reported extremely shallow divergence among several nominal species, particularly *C. macrostomum* , *C. kais* , *C. acinaces* , and *C. tenuiradius* . These findings challenge current taxonomic interpretations because some of these taxa occur in sympatry or parapatry and remain morphologically diagnosable (Freyhof, Yoğurtçuoğlu, et al. 2025). However, shallow mitochondrial divergence may arise through multiple evolutionary processes, including recent divergence, incomplete lineage sorting, historical introgression, or limited mitochondrial differentiation despite morphological and genomic divergence. Consequently, mitochondrial data alone are insufficient to fully resolve species boundaries and evolutionary relationships within the recently radiated lineages.

Genome‐wide single nucleotide polymorphism (SNP) data provide an independent source of information that can improve inference of population structure, evolutionary relationships, and lineage divergence among closely related taxa (Hashemzadeh Segherloo et al. 2021). Integrative approaches combining mitochondrial and genomic data have increasingly demonstrated that reliance on a single genetic marker may obscure evolutionary patterns and lead to misleading taxonomic conclusions (Hashemzadeh Segherloo et al. 2018, 2021; Freyhof, Hashemzadeh Segherloo, et al. 2025). For example, analyses integrating morphology, mitochondrial DNA, and genome‐wide SNPs have clarified species boundaries in several West Asian cyprinids where mitochondrial markers alone failed to capture patterns of evolutionary differentiation (Freyhof, Hashemzadeh Segherloo, et al. 2025). Despite the growing importance of genomic approaches in freshwater fish systematics, no genome‐wide assessment of taxonomic relationships within *Cyprinion* has previously been conducted.

To address this knowledge gap, we combined genotyping‐by‐sequencing (GBS) data with mitochondrial *COI* sequences to investigate the taxonomy, phylogenetic relationships, and biogeography of *C. acinaces* , *C. kais* , *C. macrostomum* , *C. microphthalmum* , *C. mhalense*, and *C. muscatense*. Specifically, we examine whether (i) currently recognized species correspond to genetically differentiated lineages, (ii) Arabian *Cyprinion* lineages share a common evolutionary origin relative to Mesopotamian and Iranian taxa, and (iii) patterns of mito‐nuclear discordance among taxa are consistent with historical introgression while also considering alternative explanations such as incomplete lineage sorting. This study illustrates how integrating mitochondrial and genome‐wide markers can improve taxonomic inferences in recently diverged freshwater fishes. The resulting framework provides new insights into the taxonomy, evolution, and biogeography of *Cyprinion* lineages across western Asia.

## 2 Materials and Methods

### 2.1 Sampling

**FIGURE 1:**
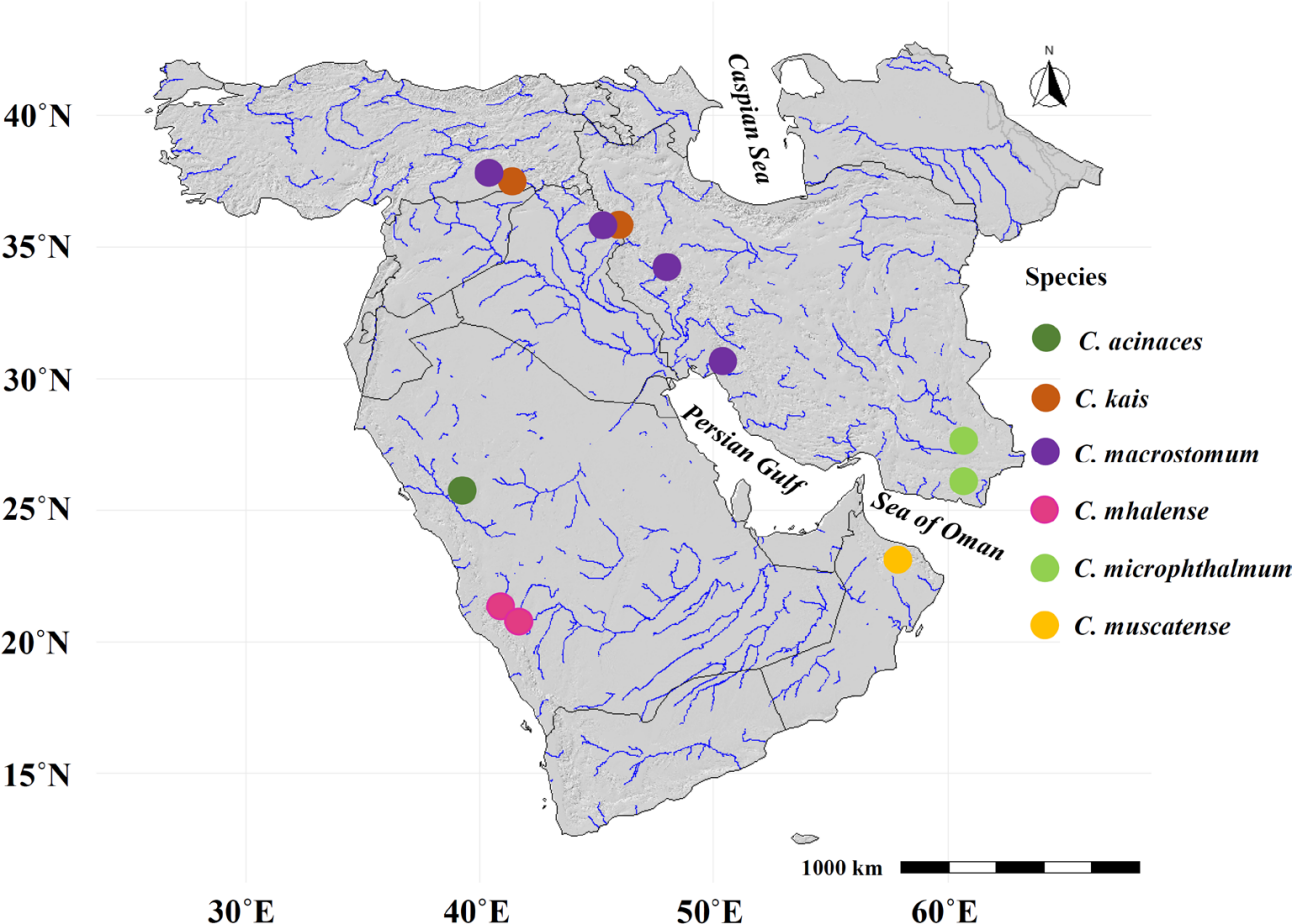
Distribution map of the species and sampling localities included in this study. Circles indicate sampling sites, and colors correspond to species as shown in the legend.

The specimens analyzed in this study included 87 samples belonging to *Cyprinion macrostomum* (total sample size [tn] = 30; sample size used for *COI* sequencing [n*COI*] = 30, sample size with SNP data [nSNP] = 7), *C. milesi* (tn = 2; n*COI* = 2, nSNP = 0), *C. kais* (tn = 3; n*COI* = 3, nSNP = 3), *C. microphthalmum* (tn = 27; n*COI* = 27, nSNP = 5), *C. acinaces* (tn = 4; n*COI* = 4, nSNP = 4), *C. mhalense* (tn = 3; n*COI* = 3, nSNP = 3), *C. muscatense* (tn = 13; n*COI* = 13, nSNP = 5), *C. tenuiradius* (tn = 3; n*COI* = 3, nSNP = 0), and *Carasobarbus luteus* (tn = 2; n*COI* = 2, nSNP = 1) as out‐group. All samples analyzed in this study were obtained from the fish and DNA collection of the Museum Koenig, Bonn, Germany and had been collected previously during legally authorized field surveys (Table SI; Figure 1). No live animals were collected specifically for the present study.

### 2.2 DNA Extraction

Genomic DNA was extracted from fin clips using the Macherey & Nagel NucleoSpin Tissue Kit following the manufacturer's protocol. Extractions were performed on an Eppendorf EpMotion automated pipetting system equipped with a vacuum manifold.

### 2.3 Mitochondrial DNA

The standard vertebrate DNA barcode region of cytochrome c oxidase subunit I (*COI*) was amplified using an *M13*‐tailed primer cocktail comprising *FishF2_t1* (5′*‐TGTAAAACGACGGCCAGTCGACTAATCATAAAGATATCGGCAC*‐3′), *FishR2_t1* (5′‐*CAGGAAACAGCTATGACACTTCAGGGTGACCGAAGAATCAGAA*‐3′), *VF2_t1* (5′‐*TGTAAAACGACGGCCAGTCAACCAACCACAAAGACATTGGCAC*‐3′), and *FR1d_t1* (5′‐*CAGGAAACAGCTATGACACCTCAGGGTGTCCGAARAAYCARAA*‐3′) (Ward et al. 2005; Ivanova et al. 2007).

PCR amplifications were carried out using Qiagen Multiplex Taq polymerase under the following conditions: initial denaturation at 95°C for 15 min; 10 touchdown cycles of 35 s at 94°C, 90 s at 52°C–49°C, and 90 s at 72°C; followed by 25 cycles of 35 s at 94°C, 90 s at 55°C, and 90 s at 72°C, with a final extension of 10 min at 72°C. PCR products were purified using ExoSAP‐IT (USB) and sequenced in both directions at Macrogen Europe Laboratories using *M13F* (5′‐*GTAAAACGACGGCCAGT*‐3′) and *M13R* (5′‐*CAGGAAACAGCTATGAC*‐3′) primers.

### 2.4 Genotyping‐By‐Sequencing (GBS)

For next‐generation sequencing, DNA concentrations were normalized to approximately 20 ng/μl to equalize read depth across samples (Hashemzadeh Segherloo et al. 2021). Genotyping‐by‐sequencing libraries were prepared following the protocols of Mascher et al. (2013) and Abed et al. (2019). Genomic DNA was digested using the restriction enzymes *PstI* and *MspI*. Digested fragments were ligated to individual‐specific oligonucleotide sequences and adapters for amplification. All individuals were multiplexed and amplified in a single reaction (Hashemzadeh Segherloo et al. 2021). Sequencing was conducted using Ion Torrent technology at the IBIS sequencing platform (Université Laval, Québec City, Canada; http://www.ibis.ulaval.ca).

### 2.5 Data Processing and Analyses

#### 2.5.1 Mitochondrial DNA

*COI* sequences were edited in BioEdit v 7.0.0 (Hall 2004), aligned in MEGA7 (Kumar et al. 2016), and inspected for stop codons. All newly generated sequences were deposited in GenBank (Table SI). Additional *Cyprinion COI* sequences were obtained from GenBank through BLAST searches (Table 1). After alignment, a 511‐bp fragment was retained for downstream analyses. The optimal nucleotide substitution model to reconstruct the mitochondrial phylogenetic relationships was selected based on the Bayesian Information Criterion (*BIC*) using ModelTest as implemented in RAxMLGUI 2.0 (Edler et al. 2021). A maximum‐likelihood (*ML*) phylogeny was reconstructed under the *HKY + G* model using RAxML. Further, a neighbor‐joining phylogenetic tree (*NJ*) was also reconstructed based on *K2P* genetic distances using MEGA7. Branch support values for *ML* and *NJ* trees were calculated using 1000 bootstrap replicates. A TCS haplotype network was constructed in PopART (Leigh et al. 2015) to visualize mutational relationships among haplotypes. Clades in haplotype network were identified based on *NJ* and *ML* clades resolved in phylogenetic trees. Two *COI* sequences of *Carassobarbus luteus* were used as outgroup.

**TABLE 1:** Details of the sequences downloaded from GenBank.

Species	Drainage	Country	Coordinates		Accession no.	Remarks
			Lon	Lat		
*Cyprinion macrostomum*	Tigris	Iran	35.2377	52.3098	KM590430	Leileh‐Sirvan River in Iran
*C. macrostomum*	Tigris	Iran	31.6738	50.7691	KM590431.1	Palangan‐Sirvan River in Iran
*C. macrostomum*	Tigris	Iran	35.2377	52.3098	KM590432.1	Leileh‐Sirvan River in Iran
*C. macrostomum*	Karun	Iran	35.2377	52.3098	KM590433.1	Armand‐Karun River in Iran
*C. macrostomum*	Tigris‐Greater Zab	Iraq	—	—	MW250387.1	—
*Cyprinion mhalense*	Al Arj Valley‐Taif	Saudi Arabia	—	—	PQ119925.1	This is *C. mhalense* from Saudi Arabia, but named as *C. watsoni* in Genbank
*Cyprinion microphthalmum*	Yazd‐Qanat	Iran	—	—	KM590429.1	The basin is Naieen‐Kerman
*Cyprinion tenuiradius*	Al Arj Valley‐Taif	Saudi Arabia	—	—	PQ119922.1	Although from Saudi Arabia, it is exactly identical (100% similarity in BLAST search) to sequence of *Cyprinion* in Marun, Shur, Zohreh, and Mond. The name in Genebank is putatively misidentified. Should be *C. tenuiradius* , but this was identified as *C. watsoni* in GenBank
*C. tenuiradius*	Al Arj Valley‐Taif	Saudi Arabia	—	—	PQ119926.1	
*C. tenuiradius*	Al Arj Valley‐Taif	Saudi Arabia	—	—	PQ119927.1	
*C. tenuiradius*	Al Arj Valley‐Taif	Saudi Arabia	—	—	PQ119928.1	
*C. tenuiradius*	Mond	Iran	28.733	51.7994	KM590434.1	Along with two other sequences identified as *C. tenuiradius* in the Mond, this sequence also from the Mond is identical to sequences from the Zohreh, Shur, and Marun rivers which had been identified as *C. macrostomum* , but based on the locality we consider this as *C. tenuiradius*
*C. tenuiradius*	Mond	Iran	28.733	51.7994	KM590435.1	
*Cyprinion watsoni*	Indus	Pakistan	—	—	PV444642.1	

#### 2.5.2 SNP Data Processing

Raw data processing, genotyping, and filtering were performed using STACKS v2.66 (Catchen et al. 2013) and the stacks_workflow pipeline v2.66 (https://github.com/enormandeau/stacks_workflow). Adapter trimming and quality filtering were conducted with Cutadapt v4.6 using parameters ‐e 0.2 and ‐m 50 (Martin 2011). Demultiplexing was performed with process_radtags (‐c ‐r ‐t 100 ‐q ‐s 0 ‐‐barcode_dist_1 2 ‐E phred33 ‐‐renz_1 PstI ‐‐renz_2 mspI).

Loci were assembled using ustacks (‐m 4 ‐M 3 ‐N 5 ‐H ‐‐deleverage), followed by cstacks (‐n 1), sstacks, tsv2bam, gstacks, and populations (‐p 1 ‐r 0.6 ‐‐fasta‐loci ‐‐vcf). This initial dataset comprised 33 samples and 146,333 SNPs, with an average genotype coverage of 19.14 and an overall missing genotype rate of 37.3%.

Subsequent SNP filtering was performed using scripts from stacks_workflow. First, SNPs were filtered (05_filter_vcf_fast.py; parameters: 3 60 0 2) to retain genotypes with a minimum coverage of three reads, a maximum of 40% missing data per group, and a minimum of two copies of the rare allele. Samples exhibiting more than 40% missing data (five individuals) were removed. The dataset was then re‐filtered (05_filter_vcf_fast.py; parameters: 3 80 0 2) to retain SNPs with ≤ 20% missing data per group. Deviant loci showing signatures of paralogy or over‐merging were identified and removed using a modified HD‐plot approach (McKinney et al. 2017; scripts 08–10 in stacks_workflow). Finally, SNPs showing highly redundant genotype information within each locus were pruned by retaining only the leftmost representative SNP per redundant pair.

ADMIXTURE v1.3.0 (Alexander et al. 2009) was used to explore population structure for *K* values ranging from 1 to 10. Due to the limited sample size, *K* = 1 was selected for missing genotype imputation. Missing genotypes were imputed by randomly drawing two alleles per locus, with allele frequencies weighted by the inferred ancestry proportions. SNP data were converted to NEXUS format using the ruby script *vcf_to_nexus.rb* (https://github.com/mmatschiner/tutorials).

##### 2.5.2.1 Population Structure

Population structure was inferred on imputed SNP data using ADMIXTURE v1.3 (different from the previous admixture analysis performed during data processing noted above) and principal component analysis (PCA). ADMIXTURE analyses were run for *K* = 1–10 with 200 bootstrap replicates. The optimal *K* was determined based on the lowest tenfold *cross‐validation* (*CV*) error. PCA was conducted using the *adegenet* R package (Jombart and Collins 2015).

To statistically evaluate clustering patterns observed in the PCA, a permutational multivariate analysis of variance (PERMANOVA) was performed on the first 10 principal components using the *adonis2* function in the *vegan* R package (Dixon 2003) with 999 permutations and Euclidean distances. Multivariate dispersion among groups was assessed using *betadisper*, followed by an ANOVA to test for heterogeneity of dispersion. Cluster robustness was further evaluated using silhouette widths calculated from k‐means clustering (*k* = 6) with the *silhouette* function in the *cluster* R package (Maechler et al. 2013).

Genomic differentiation among species was quantified using pairwise *F*_*ST*_ values calculated in R package StAMPP v1.6.3 (Pembleton et al. 2013). Statistical significance was assessed using 100 bootstrap replicates. All analyses using R packages were performed in R v 4.5.1 using RStudio 2025.09.01.

##### 2.5.2.2 Inference of Past Gene Flow

Historical gene flow was investigated using *D‐statistics* (ABBA–BABA tests) implemented in Dsuite (Malinsky et al. 2021). All possible species trios were evaluated using *C. luteus* as the outgroup. Under the null hypothesis of incomplete lineage sorting, ABBA and BABA patterns are expected to occur at equal frequencies. Following Dsuite recommendations, results with |*Z*| > 3 were considered significant evidence of introgression, whereas lower *Z* values were interpreted as suggestive evidence requiring cautious interpretation. Dsuite also calculated the *f*_4_ *‐ratio*, which estimates the proportion of admixture among taxa. Introgression signals were further localized using the *Fbranch* module, which assigns evidence of admixture to specific branches of the species tree.

##### 2.5.2.3 Species‐Tree Inference

Phylogenomic relationships were inferred using SVDquartets (Chifman and Kubatko 2014; Swofford and Kubatko 2018) as implemented in PAUP*, with *C. luteus* designated as the outgroup. All possible quartets were evaluated and assembled using the QFM quartet assembly method under the multispecies coalescent model. Branch support was assessed using 100 bootstrap replicates. In PAUP*, the “distribute” option was selected for handling ambiguous characters.

## 3 Results

### 3.1 Mitochondrial DNA

Mean interspecific Kimura two‐parameter (*K2P*) distances among the examined *Cyprinion* species ranged from 0.25% between *C. tenuiradius* and *C. macrostomum* to 12.42% between *C. milesi* and *C. muscatense* (Table 2). Genetic distances among *C. acinaces* , *C. macrostomum* , *C. kais* , and *C. tenuiradius* were particularly low, ranging from 0.25% to 0.66%.

**TABLE 2:** Mean pairwise interspecific and mean within species *K2P* sequence distances in percent (%) calculated using MEGA7 for a 511‐bp *COI* sequence.

Species	Within species	*a*	*b*	*c*	*d*	*e*	*f*	*g*	*h*	*i*
*C. acinaces* (*a*)	0.0									
*C. kais* (*b*)	0.2	0.5								
*C. macrostomum* (*c*)	0.2	0.5	0.2							
*C. mhalense* (*d*)	0.1	1.9	1.7	1.6						
*C. microphthalmum* (*e*)	1.1	3.0	2.7	2.6	2.4					
*C. milesi* (*f*)	0.0	10.9	10.7	10.6	9.7	10.2				
*C. muscatense* (*g*)	0.3	5.6	5.3	5.3	4.9	6.1	12.4			
*C. tenuiradius* (*h*)	0.1	0.6	0.4	0.2	1.8	2.4	10.5	5.4		
*C. watsoni* (*i*)	—	10.0	9.8	9.6	9.2	9.5	1.6	11.9	9.6	
*C. luteus*	0.0	16.8	16.6	16.6	16.1	16.5	15.0	17.1	16.9	14.7

**FIGURE 2:**
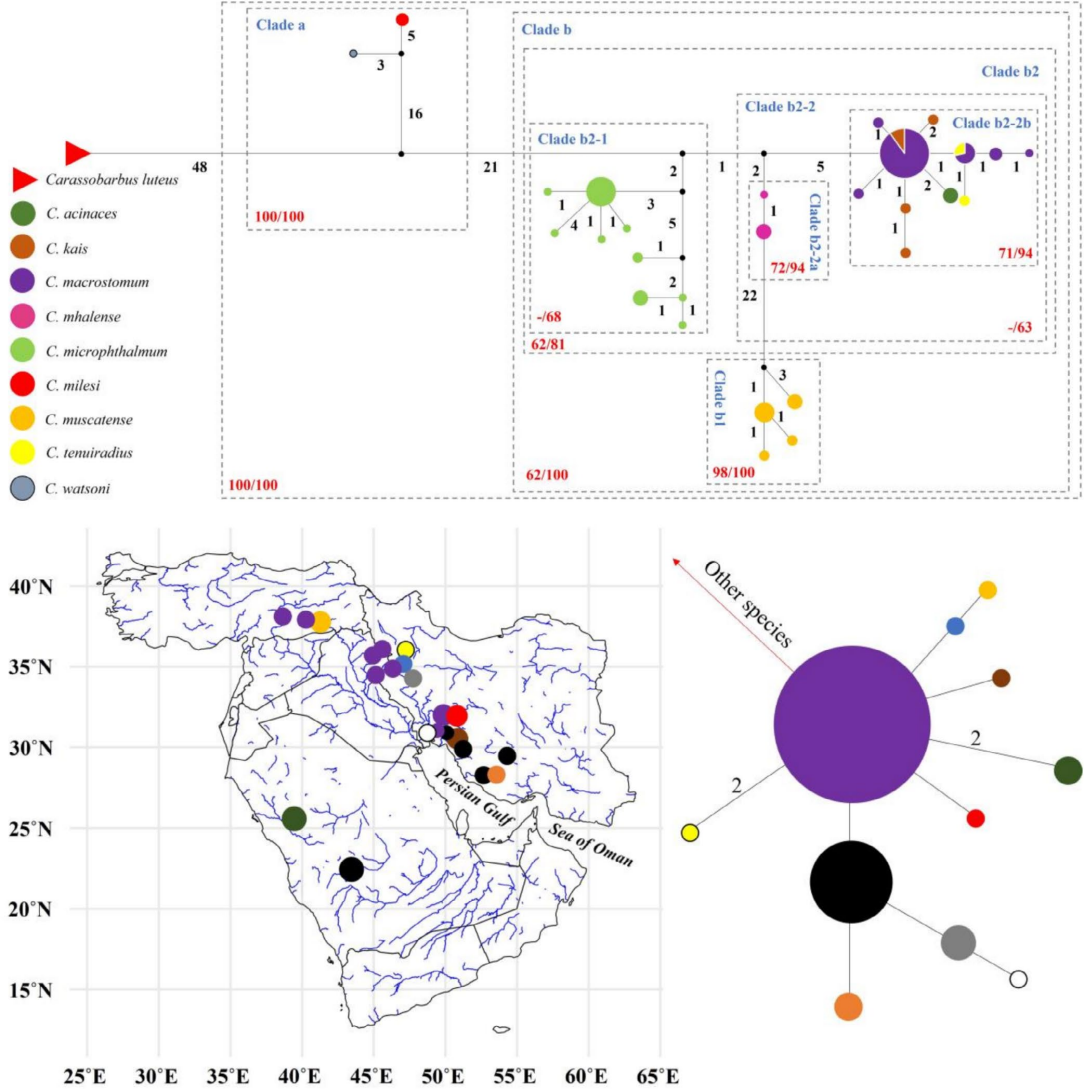
(a) Haplotype network reconstructed from a 511 bp fragment of the mitochondrial *COI* gene sequenced in this study and retrieved from GenBank. Circle sizes are proportional to haplotype frequencies. Numbers along the connecting lines indicate the number of mutational steps between haplotypes. Black circles represent inferred (hypothetical) intermediate haplotypes. Pie‐chart colors correspond to species as indicated in the legend. Gray dashed frames delineate neighbor‐joining (NJ) and maximum likelihood (ML) clades resolved among haplotypes; bootstrap support values are shown in red (ML bootstrap values before slash and NJ bootstrap values after slash). (b) Geographic distribution of haplotypes belonging to the *C. macrostomum* group (Clade b2–2b). In this panel, colors correspond only to the haplotypes shown in the network to the right of panel (b).

Both the haplotype network and phylogenetic analyses supported the monophyly of the genus *Cyprinion* relative to the outgroup taxa. However, reciprocal monophyly was not recovered for several nominal species. In particular, *C. macrostomum* , *C. kais* , *C. acinaces* , and *C. tenuiradius* shared identical or closely related mitochondrial haplotypes and formed a single mitochondrial assemblage, whereas *C. microphthalmum* , *C. mhalense*, and *C. muscatense* formed distinct haplotype groups (Figure 2). Maximum‐likelihood (*ML*) and neighbor‐joining (*NJ*) analyses recovered two major lineages within *Cyprinion* (Figure 2). *Cyprinion watsoni* formed the sister lineage to all remaining taxa (*ML*‐BS = 100; *NJ*‐BS = 100). The second lineage comprised the remaining species and was subdivided into four moderate‐ to strongly supported haplotype groups corresponding to *C. microphthalmum* , *C. mhalense*, *C. muscatense*, and the *C. macrostomum* species complex.

A total of nine closely related haplotypes were identified among *C. macrostomum* , *C. kais* , *C. acinaces* , and *C. tenuiradius* , differing by only one or two nucleotide substitutions in most cases (Figure 2). *Cyprinion kais* shared the common haplotype with *C. macrostomum* and possessed an additional haplotype differing by two base pairs (0.38% *K2P* distance). *Cyprinion tenuiradius* exhibited a haplotype identical to, or differing by a single base pair (0.19%), from the common *C. macrostomum* haplotype. The haplotype observed in *C. acinaces* differed by two base pairs (0.38%) from the predominant *C. macrostomum* haplotype. In contrast, *C. mhalense* was represented by three haplotypes and *C. muscatense* by four haplotypes. *Cyprinion microphthalmum* comprised two divergent haplotype groups containing five and four haplotypes, respectively, separated by nine nucleotide substitutions (1.74% divergence). *Cyprinion milesi* was represented by a single haplotype closely related to *C. watsoni* from Pakistan. Within the haplotype network, *C. microphthalmum* occupied an intermediate position between *C. milesi* and the remaining *Cyprinion* species.

### 3.2 Genomic Data

The final filtered dataset contained 28 samples (27 *Cyprinion* specimens and one outgroup) and 14,492 SNPs, with an average genotype coverage of 19.50 and an overall missing genotype rate of 8.59% (Supporting Information: VCF file). Sample sizes were uneven among taxa, ranging from three to seven individuals per species, and no SNP data were available for *C. tenuiradius* or *C. milesi* . Consequently, genomic inferences for some taxa should be regarded as preliminary and are interpreted with caution.

#### 3.2.1 Clustering Patterns

**FIGURE 3:**
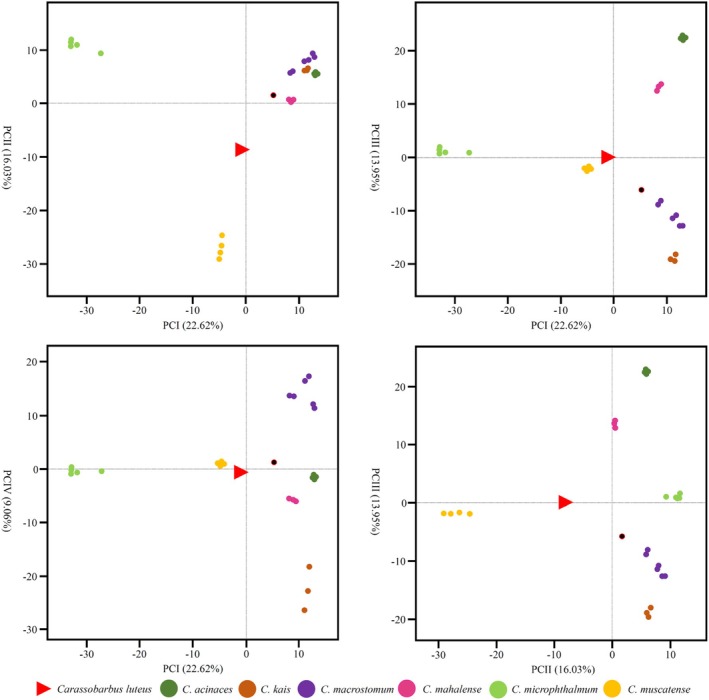
Principal component analysis (PCA) scatter plots illustrating genetic clustering of species along the first four principal component axes. Point colors correspond to species identity.

Principal component analysis (PCA) identified up to eight genetic clusters across the first four principal components, which explained 22.62%, 16.03%, 13.95%, and 9.06% of the total genomic variation, respectively (Figure 3). Two clusters corresponding to *C. microphthalmum* and *C. muscatense* were clearly separated from the remaining taxa. The remaining clusters comprised three closely located groups of *C. macrostomum* together with distinct clusters corresponding to *C. kais* , *C. mhalense*, and *C. acinaces* . Although these clusters were positioned relatively close to one another in PCA space, no overlap among species was observed for the individuals analyzed. One *C. macrostomum* specimen from the Marun River drainage was displaced toward *C. muscatense* and *C. mhalense* along the first two principal components. PERMANOVA performed on the first 10 PC axes indicated that species identity explained a significant proportion of the genomic variation (*R* ^2^ = 0.89, *F* = 22.86, *p* < 0.01). Multivariate dispersion analyses also revealed significant differences among species (*F* = 3.66, *p* < 0.05). Silhouette analyses of *k*‐means clustering indicated generally strong assignment of individuals to clusters (mean silhouette width = 0.69). Four clusters showed very strong support (average silhouette width > 0.85), whereas smaller clusters exhibited moderate support (0.21–0.56).

**FIGURE 4:**
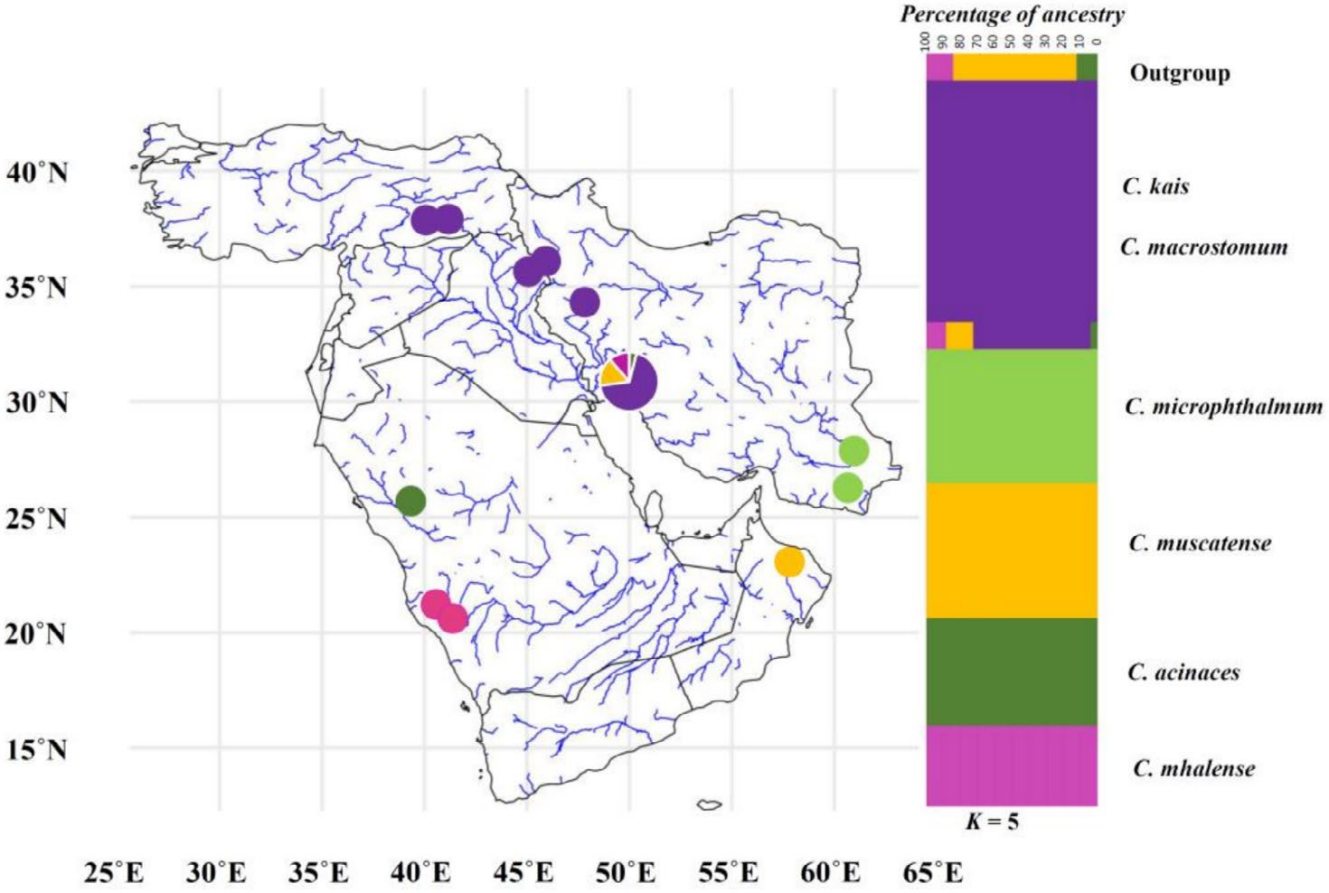
ADMIXTURE results (*K* = 5) displayed as pie charts overlaid on a geographic map. Each pie chart represents the ancestry proportions of an individual, with colors corresponding to inferred genomic clusters. The full ADMIXTURE bar plot, including the outgroup (uppermost individual), is shown to the right of the map. Pie charts for individuals showing admixture (introgression) are displayed at a larger size for visibility; pie chart size does not reflect sample size.

ADMIXTURE analyses identified five genomic clusters as the most likely population structure based on cross‐validation error values (Figure 4). Under this model, *C. macrostomum* and *C. kais* shared a common ancestry, whereas *C. acinaces* , *C. mhalense*, *C. microphthalmum* , and *C. muscatense* each formed distinct genomic clusters. One *C. macrostomum* individual from the Marun River drainage exhibited mixed ancestry associated primarily with *C. muscatense*, *C. acinaces* , and *C. mhalense*.

Pairwise *F*_ST_ estimates revealed significant nuclear differentiation among all examined species (*p* < 0.001; Table SII). The lowest differentiation occurred between *C. kais* and *C. macrostomum* (*F*_ST_ = 0.316). Although pairwise *F*_ST_ values were relatively high, these estimates should be interpreted cautiously because of small and uneven sample sizes among taxa, which may reduce the precision of differentiation estimates (Meirmans and Hedrick 2011; Willing et al. 2012).

**FIGURE 5:**
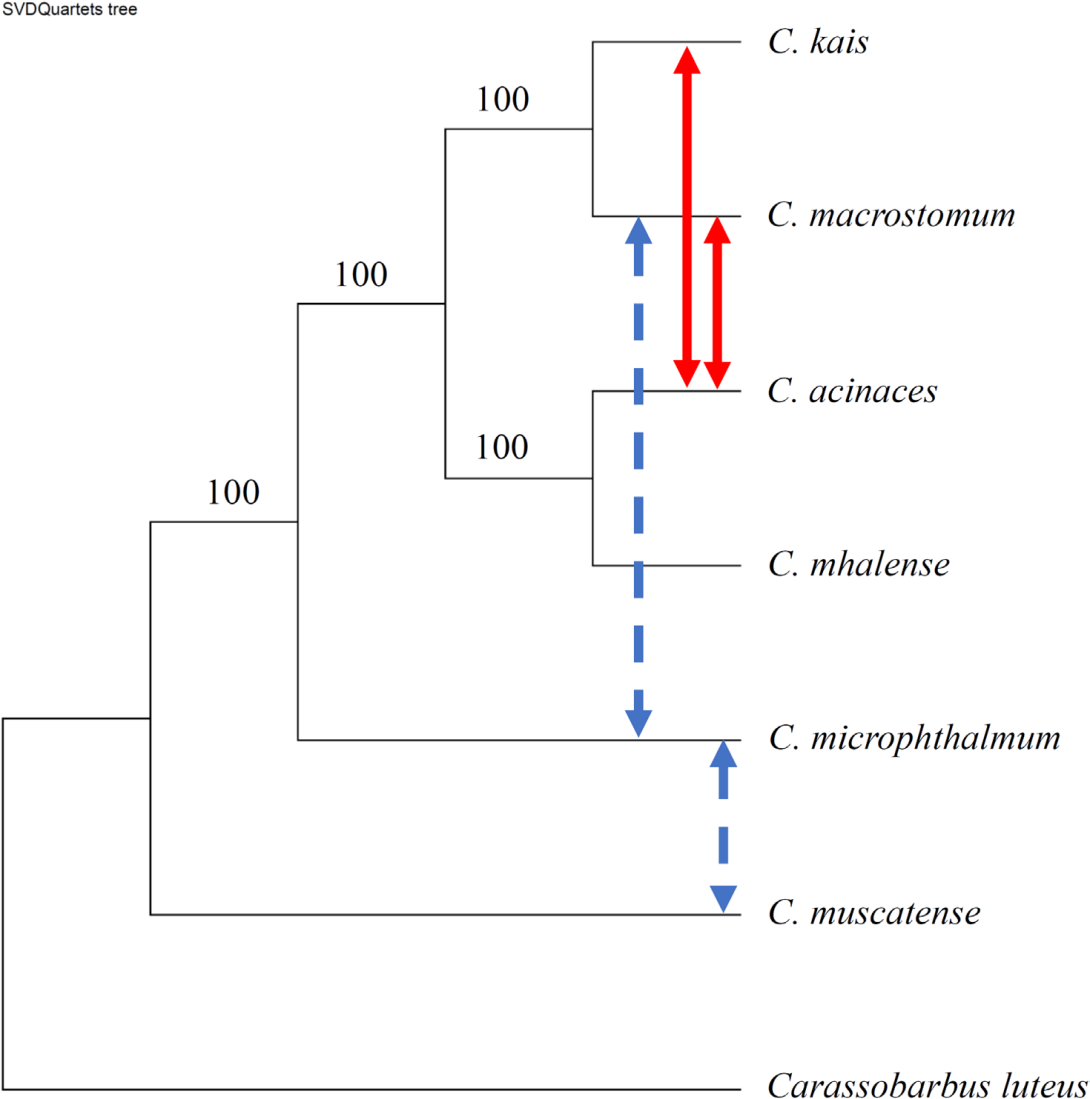
Species tree inferred using SVDQuartets based on SNP data, with *Carassobarbus luteus* used as the outgroup. Numbers along branches indicate bootstrap support values. Arrows between branches denote gene flow inferred from f‐branch analyses: Red arrows indicate strong introgression signals, whereas blue dashed arrows indicate moderate or weak signals.

The coalescent‐based species tree recovered a strongly supported clade containing *C. macrostomum* , *C. kais* , *C. acinaces* , *C. mhalense*, and *C. microphthalmum* (BS = 100; Figure 5). Within this clade, *C. macrostomum* and *C. kais* formed a strongly supported sister group (BS = 100), whereas *C. acinaces* and *C. mhalense* formed a second strongly supported lineage (BS = 100). *Cyprinion microphthalmum* was recovered as the sister lineage to the latter clade.

#### 3.2.2 Signatures of Gene Flow

*D‐statistic* analyses identified strong evidence of introgression between *C. macrostomum* and *C. acinaces* (*D* = 0.184, *Z* = 4.84, *p* < 0.001; Table 3). Several additional comparisons yielded elevated *D‐statistics*, including *C. acinaces* – *C. kais* (*D* = 0.117, *Z* = 2.77) and *C. microphthalmum* –*C. muscatense* (*D* = 0.156, *Z* = 2.67). However, because these comparisons did not exceed the predefined significance threshold (|*Z*| > 3), they are interpreted as suggestive rather than statistically significant evidence of introgression. Consistent with the *D‐statistic* results, *f‐branch* analyses identified signals of historical gene flow involving *C. macrostomum* and *C. acinaces* , *C. microphthalmum* and *C. muscatense*, and *C. kais* and *C. acinaces* .

**TABLE 3:** Highly supported and moderate signatures of gene flow calculated with Dsuite.

P1	P2	P3	D	Z‐score	*p*	f4‐ratio
*C. mhalense*	*C. acinaces*	*C. macrostomum*	0.184	4.836	1.3e‐06	0.148
*C. kais*	*C. macrostomum*	*C. acinaces*	0.130	4.339	1.4e‐05	0.088
*C. mhalense*	*C. acinaces*	*C. kais*	0.117	2.767	0.005	0.047
*C. acinaces*	*C. microphthalmum*	*C. muscatense*	0.156	2.669	0.007	0.087
*C. mhalense*	*C. macrostomum*	*C. microphthalmum*	0.105	2.163	0.030	0.041
*C. macrostomum*	*C. microphthalmum*	*C. muscatense*	0.097	2.154	0.031	0.059
*C. acinaces*	*C. macrostomum*	*C. muscatense*	0.071	2.094	0.036	0.029

*Note:* The gray highlight denotes highly significant (*Z* > 3) gene flow.

## 4 Discussion

This study provides the first genome‐wide assessment of taxonomic and biogeographic relationships within *Cyprinion*. By integrating mitochondrial DNA and genome‐wide SNP data, we demonstrate that several nominal species exhibit clear genomic differentiation despite sharing identical or closely related mitochondrial haplotypes. These results highlight the complexity of species boundaries within the genus and illustrate the limitations of relying exclusively on mitochondrial markers when evaluating recently diverged lineages.

Several conclusions presented here should be interpreted cautiously due to small sample sizes. Although the final SNP dataset comprised 14,492 loci, individuals used in genomic analysis were restricted to 27 *Cyprinion* individuals distributed unevenly among species, with some taxa represented by only three to five individuals. In addition, no SNP data were available for *C. tenuiradius* or *C. milesi* . Consequently, the genomic distinctiveness and evolutionary relationships inferred for some taxa should be regarded as preliminary pending a wider geographic and larger genomic sampling. Further, we have used different clustering and phylogenetic approaches, which differ in their background models. These approaches do not always produce identical patterns; hence, concordant patterns from multiple analyses are interpreted as stronger evidence of differentiation than the results of any single method alone.

### 4.1 Taxonomic Implications

The mitochondrial data do not support reciprocal monophyly of several currently recognized *Cyprinion* species: *C. macrostomum* , *C. kais* , *C. acinaces* , and *C. tenuiradius* share identical or closely related haplotypes and form a single mitochondrial assemblage. Similar patterns have been reported previously by Durand et al. (2002) and Esmaeili et al. (2024). Such shallow mitochondrial divergence may reflect several non‐exclusive processes, including recent divergence (Jacobsen et al. 2012), incomplete lineage sorting (DeRaad et al. 2023), introgression, or taxonomic over‐splitting. Consequently, mitochondrial data alone are insufficient to discriminate among these alternative explanations.

Although *D*‐statistic analyses and mito‐nuclear discordance are consistent with historical introgression among some lineages, the present dataset does not allow definitive discrimination between introgression and incomplete lineage sorting. Given the shallow divergence among several taxa, both processes may have contributed to the observed patterns. In the following passages, taxonomy of the *C. macrostomum* species complex and other distinct species of the genus will be discussed, integrating morphological, mitochondrial, and genomic evidence.

#### 4.1.1 *Cyprinion macrostomum* Species Complex

The shallow mitochondrial divergence observed among *C. macrostomum* , *C. kais* , *C. acinaces* , and *C. tenuiradius* contrasts with their morphological differentiation and, where available, genomic evidence. These taxa therefore represent the principal source of taxonomic uncertainty within the genus and illustrate the importance of integrating multiple datasets when evaluating species boundaries.

*Cyprinion acinaces* is endemic to Saudi Arabia and Yemen and is currently allopatric from *C. macrostomum* , *C. kais* , and *C. tenuiradius* (Freyhof, Yoğurtçuoğlu, et al. 2025; for taxonomic details see Table SIII). Two population groups are currently recognized: *C. a. hijazi*, inhabiting headwaters of the Hijaz Mountains in Saudi Arabia, and *C. a. acinaces*, endemic to the Hadramaut region of Yemen (Freyhof, Yoğurtçuoğlu, et al. 2025). Alotaibi et al. (2020) suggested that these populations may represent a single species. However, whether the two forms are conspecific remains unresolved because comprehensive comparative genomic and morphological data are currently unavailable. The material examined in the present study, as well as that analyzed by Esmaeili et al. (2024), belongs to *C. a. hijazi*.

Despite the large geographic gap separating *C. acinaces* from species occurring in the Persian Gulf basin, it exhibits high similarity to Persian Gulf taxa in both mitochondrial and nuclear DNA. This pattern may reflect a relatively recent biogeographic separation from ancestral populations inhabiting formerly connected drainage systems, although alternative historical scenarios cannot be excluded.

Although mitochondrial evidence alone does not support clear species‐level separation within the *C. macrostomum* species complex, genome‐wide analyses consistently recovered *C. acinaces* and *C. macrostomum* as distinct but genomically similar lineages, suggesting that taxonomic over‐splitting alone may not fully explain the observed pattern. In addition, *C. acinaces* possesses private mitochondrial haplotypes that are similar to, but not identical with, those of *C. macrostomum* . According to Freyhof, Yoğurtçuoğlu, et al. (2025), both species share an arched mouth with a cornified sheet on the lower jaw and a long, strongly ossified last unbranched dorsal‐fin ray. However, *C. macrostomum* typically possesses a slightly higher number of branched dorsal‐fin rays than *C. acinaces* , whereas detailed information on the morphology and nuptial colouration of *C. acinaces* remains limited. The observed genetic structuring, together with their geographic isolation, provides preliminary support for the recognition of *C. acinaces* as a separate evolutionary lineage. Nevertheless, broader geographic and genomic sampling, combined with reassessment of morphological characters and nuptial colouration, will be required to fully evaluate alternative taxonomic interpretations.

*Cyprinion tenuiradius* remains the most taxonomically uncertain. This species occupies coastal drainages of the Persian Gulf basin between the distributions of *C. macrostomum* and *C. microphthalmum* and exhibits morphological features intermediate between these species (Freyhof, Yoğurtçuoğlu, et al. 2025). Three of the five individuals examined from the Mond River shared mitochondrial haplotypes with *C. macrostomum* , whereas the remaining two individuals possessed a closely related haplotype differing by only a single nucleotide substitution. Freyhof, Yoğurtçuoğlu, et al. (2025) proposed that populations currently assigned to *C. tenuiradius* may represent hybrid populations originating from *C. macrostomum* and *C. microphthalmum* . Given its geographic position between these species, this hypothesis is possible. However, alternative explanations, including incomplete lineage sorting and recent divergence, remain equally possible. Overall, no genome‐wide SNP data were available for *C. tenuiradius* in the present study. Consequently, all conclusions regarding its evolutionary history and taxonomic status await confirmation in follow‐up surveys. Additional genome‐wide sampling across its distribution range will be required to evaluate these competing hypotheses.

**FIGURE 6:**
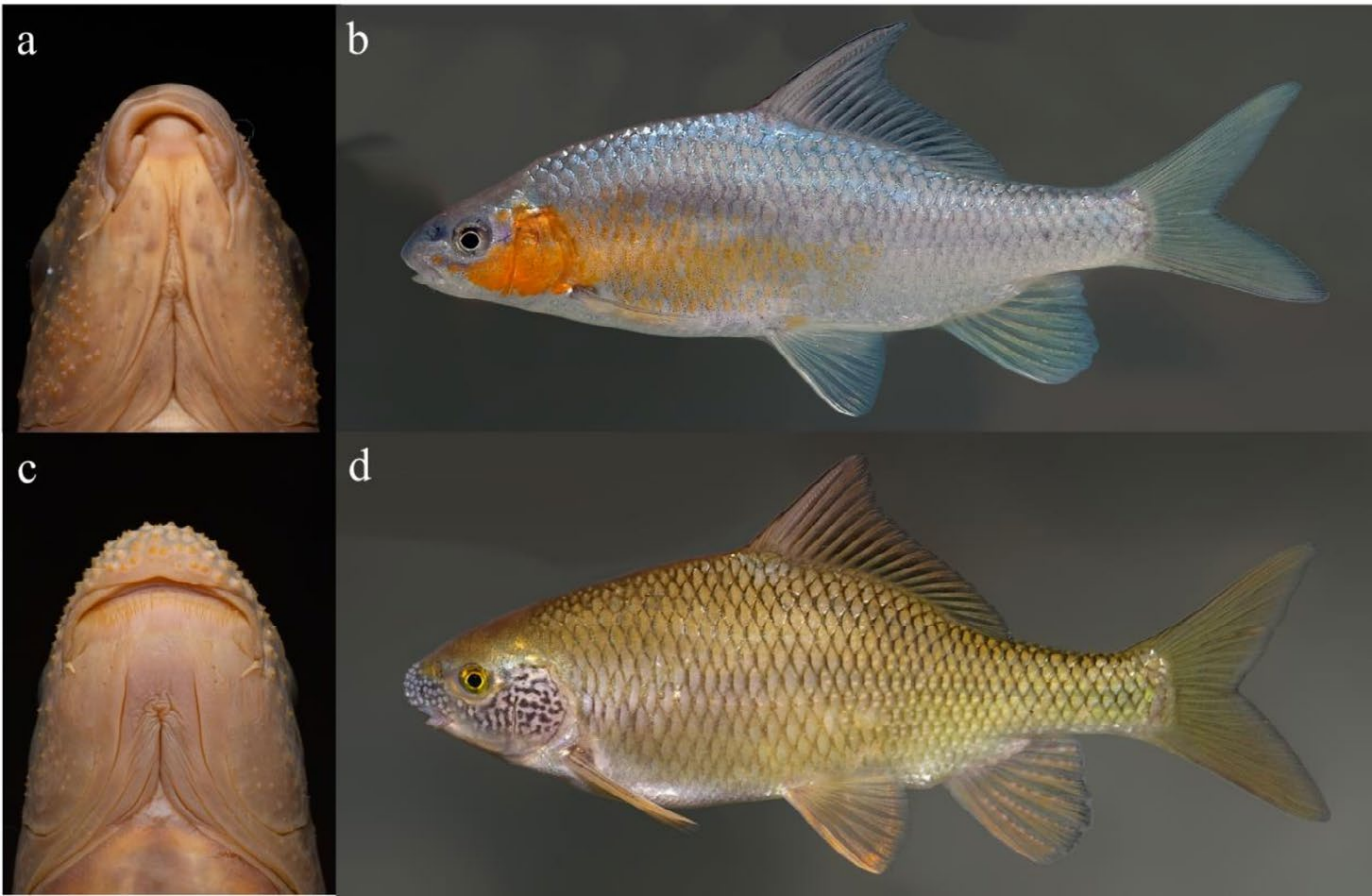
Mouth and body features in *C. kais* (a, b) versus *C. macrostomum* (c, d). (a) *C. kais* (FSJF 2867, Batman River, Türkiye; male, 118 mm SL), (b) *C. kais* (Lesser Zab drainage, Iraq; male, ~100 mm SL, male), (c) *C. macrostomum* (FSJF 3416, Lesser Zab drainage, Iraq; male, 135 mm SL), and (d) *C. macrostomum* (Lesser Zab drainage, Iraq; 150 mm SL). J. Freyhof.

*Cyprinion kais* shares identical mitochondrial haplotypes with *C. macrostomum* but also possesses closely related private haplotypes. Despite this mitochondrial similarity, the two taxa differ markedly in ecology, morphology, and male nuptial colouration (Freyhof, Yoğurtçuoğlu, et al. 2025; Figure 6). Whereas *C. macrostomum* is ecologically widespread and occurs in a broad range of habitats, *C. kais* is largely restricted to fast‐flowing middle sections of larger rivers and exhibits a considerably narrower ecological niche.

The shallow mitochondrial divergence despite morphological and genomic divergence between these taxa may reflect very recent diversification, incomplete lineage sorting, or historical introgression. The present dataset does not allow definitive discrimination among these alternatives. Furthermore, different genomic analyses provide partially different perspectives on their relationship. ADMIXTURE grouped both taxa within the same ancestry component at the optimal *K* value, but PCA, and pairwise *F*_ST_ analyses consistently detected significant nuclear differentiation between them (*F*_ST_ = 0.316, *p* < 0.001). The observed nDNA differentiation suggests some degree of evolutionary independence between these taxa. Hence, this evolutionary differentiation, together with their pronounced differences in mouth morphology and male nuptial colouration, provides preliminary support for the recognition of *C. kais* as a distinct evolutionary lineage. One possible explanation for the observed morphological divergence despite limited mitochondrial differentiation is character displacement following secondary contact (Robinson and Wilson 1994; Hashemzadeh Segherloo et al. 2017). However, the present study does not directly test this mechanism, and alternative explanations remain possible. The restriction of diagnostic colouration to males may also indicate the involvement of sex‐linked or sex‐influenced traits, although this hypothesis requires dedicated genomic and common‐garden experiments. More generally, the present dataset does not include ecological, morphometric data, and therefore hypotheses involving character displacement, ecological divergence (Levin et al. 2021; Komarova et al. 2022; Hashemzadeh Segherloo, Najafi Chaloshtory, et al. 2022, 2023), or the involvement of specific genomic regions (Poelstra et al. 2014) should be regarded as preliminary explanations requiring further investigation.

*Cyprinion mhalense* occurs in the eastern Sarawat Mountains of the Arabian Peninsula, and is allopatric to *C. acinaces* , which inhabits the western and southern coastal drainages of the Arabian Peninsula. The two species are morphologically distinguishable based on multiple meristic characters (Freyhof, Yoğurtçuoğlu, et al. 2025). Mitochondrial haplotypes of *C. mhalense* form a distinct clade that is sister to the *C. macrostomum* species complex. With *K2P* distances of 1.68%–1.95% from members of this group, *C. mhalense* occupies an intermediate position between the more divergent Arabian taxa and the closely related *C. macrostomum* species complex. Similarly, PCA and species‐tree analyses place *C. mhalense* relatively close to *C. acinaces* and the other members of *C. macrostomum* species complex. One possible explanation for this pattern is historical genetic exchange involving lineages related to *C. tenuiradius* . Several *COI* sequences from the Al Arj Valley in Saudi Arabia (e.g., GenBank accessions PQ119922, PQ119926‐PQ119928) have been identified as either *C. mhalense* or *C. tenuiradius* , suggesting a complex evolutionary history involving lineages currently distributed on opposite sides of the Persian Gulf. However, because genome‐wide SNP data are currently unavailable for *C. tenuiradius* , this hypothesis cannot be evaluated directly and should be regarded as tentative.

Overall, the allopatric distribution of *C. mhalense* within the Arabian Peninsula, its clear morphological distinctiveness, and its consistent genomic differentiation support its recognition as a valid species. At the same time, its phylogenetic position suggests a close evolutionary relationship with members of the *C. macrostomum* species complex that warrants further investigation using wider geographic and genomic sampling.

#### 4.1.2 Other Taxa

Although *C. milesi* was not included in the genomic analyses, mitochondrial evidence indicates a relatively close relationship with *C. watsoni* , consistent with the morphological data reported by Freyhof, Yoğurtçuoğlu, et al. (2025). *Cyprinion milesi* occurs sympatrically with *C. microphthalmum* and geographically close to the distribution range of *C. watsoni* . Despite its close mitochondrial relationship with *C. watsoni* , the approximately 1.5% *K2P* divergence between these taxa and their reported morphological differences suggest that their taxonomic status remains uncertain. Consequently, additional integrative studies incorporating genome‐wide markers and detailed morphological analyses will be necessary to clarify their evolutionary relationships and taxonomic boundaries.

Among the species examined in this study, *C. muscatense* and *C. microphthalmum* exhibit the strongest evidence for evolutionary distinctiveness. Both species are consistently differentiated from each other and from other species across mitochondrial phylogenies, PCA, ADMIXTURE analyses, species‐tree inference, and pairwise genetic differentiation estimates. These concordant lines of evidence provide strong support for their recognition as distinct evolutionary lineages, also reported in Freyhof, Yoğurtçuoğlu, et al. (2025).

### 4.2 Biogeographic Considerations

The biogeographic scenarios discussed below should be regarded as hypotheses that are consistent with the observed mitochondrial and genomic patterns rather than formally tested historical reconstructions. The present study does not incorporate divergence‐time estimation, ancestral‐range reconstruction, demographic modeling, or paleohydrological analyses and therefore cannot easily discriminate among alternative historical scenarios.

Banarescu and Herzig‐Straschil (1995) proposed two major biogeographic groups within *Cyprinion*: (1) a *C. macrostomum* group distributed across the Quweiq, Tigris‐Euphrates, and western Iranian drainages, and (2) a *C. watsoni–C. microphthalmum* group extending across southern Arabia, southern and central Iran, Afghanistan, and western Pakistan. The results of this study generally support this framework, but refine several relationships revealed by both mitochondrial and genomic data.

The combined evidence identifies a broad clade comprising *C. macrostomum* , *C. kais* , *C. acinaces* , *C. tenuiradius* , and *C. mhalense*, extending from Mesopotamia and southern Iran into the Arabian Peninsula. In contrast, *C. milesi* shares closely related mitochondrial haplotypes with *C. watsoni* , supporting their placement within a southeastern assemblage. *Cyprinion microphthalmum* forms a distinct lineage that is more closely related to the *C. macrostomum* species complex than to *C. watsoni* or *C. muscatense*, whereas *C. muscatense* represents a deeply differentiated lineage restricted to the Arabian Peninsula. These mitochondrial relationships are broadly consistent with the genomic clustering patterns recovered from the SNP dataset. Based on these findings, four principal biogeographic assemblages can be recognized within *Cyprinion*:

- a *C. watsoni* – *C. milesi* group distributed from southeastern Iran (Sarbaz and Mashkid drainages) eastward to the Indus basin in Pakistan;
- a *C. microphthalmum* group extending from the Shur River eastward into the Makran region and occupying several endorheic basins, including Yazd (Abarghou‐Sirjan), Jazmurian, Mashkid, Gonabad, and the Lut Desert;
- a *C. macrostomum* species complex comprising *C. macrostomum* , *C. kais* , *C. tenuiradius* , *C. acinaces* , and *C. mhalense*, ranging from Mesopotamia eastward to southern Iranian drainages and southward into Arabian drainages; and
- *C. muscatense*, restricted primarily to wadis of the Hajar Mountains in Oman and the United Arab Emirates.

The extremely shallow mitochondrial divergence among *C. macrostomum* , *C. kais* , *C. acinaces* , and *C. tenuiradius* is consistent with relatively recent dispersal or vicariant events linking Mesopotamian, Iranian, and Arabian populations. Comparable dispersal scenarios have previously been proposed for other West Asian freshwater fishes, including *Garra rufa* , whose distribution has been linked to palaeodrainage systems associated with the exposed Persian Gulf basin during periods of lowered sea level (Rose 2010; Groucutt and Petraglia 2012; Fagan 2014; Hashemzadeh Segherloo et al. 2017, 2026).

One possible explanation for the particularly close relationship between *C. acinaces* and members of the *C. macrostomum* species complex involves the palaeodrainage system commonly referred to as the Pishon River (Holm 1960; Sauer 1996; Parker and Rose 2008; Matter et al. 2016). This river has been interpreted as a large drainage network connecting parts of the Hijaz region of western Arabia to the northern Persian Gulf through the Wadi al‐Batin and Wadi al‐Rummah systems. If such a connection persisted during periods of higher precipitation, it could have facilitated freshwater dispersal between Mesopotamia and western Arabia.

Under this scenario, ancestral populations related to the present‐day *C. macrostomum* species complex may have dispersed through the Pishon palaeodrainage before becoming isolated following its desiccation. Present‐day populations of *C. acinaces* in the Hijaz Mountains could therefore represent remnants of a former biogeographic connection between Mesopotamia and western Arabia. The shallow mitochondrial divergence observed between *C. acinaces* and members of the *C. macrostomum* species complex is consistent with such a recent separation. However, because the present study lacks temporal calibration and demographic analyses, this hypothesis should be regarded as one of several plausible historical scenarios rather than a demonstrated biogeographic reconstruction.

It is also noteworthy that additional GenBank sequences from the Al Arj Valley near Taif in Saudi Arabia are identical to haplotypes recorded from the Mond River in Iran. This pattern is consistent with the existence of relatively recent historical connections among Arabian and Persian Gulf drainages.

Future studies incorporating wider geographic sampling, genome‐wide data from currently unsampled taxa, divergence‐time estimation, and demographic modeling approaches such as Approximate Bayesian Computation could provide more rigorous tests of alternative dispersal and divergence scenarios within *Cyprinion*.

## 5 Conclusion

This study provides the first genome‐wide assessment of taxonomic and biogeographic relationships within the genus *Cyprinion*. By integrating mitochondrial DNA and genome‐wide SNP data, we demonstrate that several closely related taxa exhibit substantial genomic differentiation despite sharing identical or closely related mitochondrial haplotypes. These findings show that shallow mitochondrial divergence does not necessarily indicate a lack of evolutionary differentiation and highlight the limitations of relying exclusively on mitochondrial markers for species delimitation in recently diverged lineages.

The strength of evidence supporting currently recognized species varies among taxa. *Cyprinion muscatense* and *C. microphthalmum* are consistently differentiated in both mitochondrial and genomic analyses and therefore represent well‐supported evolutionary lineages. *Cyprinion kais* and *C. acinaces* also show clear nuclear DNA differentiation despite shallow mitochondrial divergence, providing preliminary support for their recognition as distinct lineages. In contrast, the taxonomic status of *C. tenuiradius* and *C. milesi* remains unresolved because genome‐wide SNP data are currently unavailable for these taxa.

Overall, the discordance observed between mitochondrial and genomic datasets highlights the importance of integrative approaches combining morphology, mitochondrial DNA, and genome‐wide markers when evaluating species boundaries and evolutionary relationships in recently diverged freshwater fishes. Future studies incorporating broader geographic sampling, larger genomic datasets, demographic modeling, and detailed morphological analyses will be essential for resolving the remaining taxonomic and biogeographic uncertainties within *Cyprinion*.

## Author Contributions

**Iraj Hashemzadeh Segherloo:** conceptualization (leading), data curation (equal), formal analysis (leading), funding acquisition (leading), resources (equal), writing – original draft (leading), writing – review and editing (leading). **Matthias F. Geiger:** data curation (leading), formal analysis (equal), writing – original draft (equal), writing – review and editing (equal). **Eric Normandeau:** data curation (leading), formal analysis (leading), writing – original draft (equal), writing – review and editing (equal). **Jörg Freyhof:** conceptualization (leading), writing – original draft (equal), writing – review and editing (equal).

## Funding

This work was supported by Shahrekord University (688MIGRD94), and Alexander von Humboldt‐Stiftung, Bonn, Germany.

## Conflicts of Interest

The authors declare no conflicts of interest.

## Supporting information


**Table SI:** Details of individuals used for *COI* and SNP analyses in this study. Y: available, N: not available.

**Table SII:** Pairwise *F*_ST_ values among *Cyprinion* species calculated from 14,492 SNPs. *F*_ST_ values are shown below the diagonal, and associated *p*‐values are shown above the diagonal.

**Table SIII:** Chronological order of currently recognized species of Cyprinion, including original combinations, authorship, and type localities. Species originally described under other genera (e.g., *Scaphiodon* and *Barbus*) are indicated by their original combinations.

**Data S1:** Supporting_SNP_data.

## Data Availability

The VCF file used for the analyses is available as a Supporting Information to the publication. The *COI* sequences produced in this study can be accessed from GenBank using accession numbers: PZ165845‐PZ165913 and PZ019771‐PZ019789.
