# Supplementary material for: Mito‐Nuclear Discordance and Species Boundaries in the Freshwater Fish Genus Cyprinion Revealed by Genome‐Wide SNPs
Source: Ecol Evol. 2026 Jul 31;16(8):e73893. doi: 10.1002/ece3.73893 (PMC13426011; doi:10.1002/ece3.73893)
Supplement: Supplementary file 1 — Table SI: Details of individuals used for COI and SNP analyses in this study. Y: available, N: not available. [file ECE3-16-e73893-s002.docx]

Supplementary Table I. Details of individuals used for *COI* and SNP analyses in this study. Y: available, N: not available.

| Museum Sample ID | Country | Drainage | Species | Identifier | Data available | | GenBank accession no. | Coordinates | |
| --- | --- | --- | --- | --- | --- | --- | --- | --- | --- |
|  |  |  |  |  |  |  |  | lat | long |
|  |  |  |  |  | mtDNA | SNP |  |  |  |
| 24IJFDNA3796 | Iran | Tigris | *Cyprinion macrostomum* | - | Y | N | PZ165845 | 33.463.587 | 46.477.451 |
| 24IJFDNA3815 | Iran | Karkheh-Tigris | *Cyprinion macrostomum* | - | Y | N | PZ165847 | 34.421.452 | 47.890.665 |
| 24IJFDNA3825 | Iran | Tigris | *Cyprinion macrostomum* | - | Y | N | PZ165848 | 34.475.362 | 45.580.317 |
| 24IJFDNA3837 | Iran | Tigris | *Cyprinion macrostomum* | - | Y | N | PZ165849 | 33.905.467 | 46.169.241 |
| 24IJFDNA3840 | Iran | Tigris | *Cyprinion macrostomum* | - | Y | N | PZ165850 | 32.822.234 | 46.974.764 |
| 24IJFDNA3862 | Iran | Mond | *Cyprinion tenuiradius* | - | Y | N | PZ165851 | 28.721.312 | 52.418.881 |
| 24IJFDNA3865 | Iran | Tigris | *Cyprinion macrostomum* | - | Y | N | PZ165852 | 36.358.573 | 45.421.733 |
| 24IJFDNA3879 | Iran | Jazmurian | *Cyprinion microphtalmum* | - | Y | N | PZ165853 | 27.851.609 | 60.762.450 |
| 2194aCEsinII19 | Iran | Makran | *Cyprinion milesi* | - | Y | N | PZ165854 | 26.259.578 | 60.756.800 |
| 2194bCEsinII19 | Iran | Makran | *Cyprinion milesi* | - | Y | N | PZ165855 | 26.259.578 | 60.756.800 |
| 3234JF-IGB01 | Oman | Hatta | *Cyprinion muscatense* | - | Y | N | PZ165856 | 24.712.948 | 56.186.198 |
| 3242JF-IGB01 | Oman | Kabbah | *Cyprinion muscatense* | - | Y | N | PZ165857 | 2.302.213 | 5.872.631 |
| 3252JF-IGB01 | Iran | Makran | *Cyprinion microphthalmum* | - | Y | N | PZ165858 | 26.259.578 | 60.756.800 |
| 3253JF-IGB01 | Iran | Mashkid | *Cyprinion microphthalmum* | - | Y | N | PZ165859 | 27.367.758 | 61.303.247 |
| 3254JF-IGB01 | Iran | Mashkid | *Cyprinion microphthalmum* | - | Y | N | PZ165860 | 27.367.758 | 61.303.248 |
| 3255JF-IGB01 | Iran | Bedjestan | *Cyprinion microphthalmum* | - | Y | N | PZ165861 | 34.332.222 | 58.683.925 |
| 3257JF-IGB01 | Iran | Makran | *Cyprinion microphthalmum* | - | Y | N | PZ165862 | 26.440.922 | 60.158.650 |
| 3258JF-IGB01 | Iran | Mashkid | *Cyprinion microphthalmum* | - | Y | N | PZ165863 | 27.143.392 | 63.242.369 |
| 3259JF-IGB01 | Iran | Jazmurian | *Cyprinion microphthalmum* | - | Y | N | PZ165864 | 27.184.764 | 60.585.281 |
| 3260JF-IGB01 | Iran | Mashkid | *Cyprinion microphthalmum* | - | Y | N | PZ165865 | 28.513.858 | 61.524.936 |
| 3261JF-IGB01 | Iran | Makran | *Cyprinion microphthalmum* | - | Y | N | PZ165866 | 26.440.922 | 60.158.650 |
| 3263JF-IGB01 | Iran | Jazmurian | *Cyprinion microphthalmum* | - | Y | N | PZ165867 | 27.184.764 | 60.585.281 |
| 3264JF-IGB01 | Iran | Mashkid | *Cyprinion microphthalmum* | - | Y | N | PZ165868 | 28.513.858 | 61.524.936 |
| Museum Sample ID | Country | Drainage | Species | Identifier | Data available | | GenBank accession no. | Coordinates | |
|  |  |  |  |  | mtDNA | SNP |  | lat | long |
| Caraso2 | Iran | Jarahi | *Carasobarbus luteus* | Caraso2 | Y | Y | PZ165869 | 30.868.795 | 5.016.194 |
| Caraso5 | Iran | Jarahi | *Carasobarbus luteus* | Caraso5 | Y | N | PZ165870 | 30.868.795 | 5.016.194 |
| cn7581 | UAE | Shuwayhah | *Cyprinion muscatense* | - | Y | N | PZ165871 | - | - |
| cn7681 | UAE | Shuwayhah | *Cyprinion muscatense* | - | Y | N | PZ165872 | - | - |
| cn7688 | UAE | Shuwayhah | *Cyprinion muscatense* | - | Y | N | PZ165873 | - | - |
| CyprGam1 | Iran | Karkheh | *Cyprinion macrostomum* | CyprGam1 | Y | Y | PZ165874 | 34.388.196 | 47.707.334 |
| CyprGam2 | Iran | Karkheh | *Cyprinion macrostomum* | CyprGam2 | Y | Y | PZ165875 | 34.388.196 | 47.707.334 |
| CyprGam3 | Iran | Karkheh | *Cyprinion macrostomum* | CyprGam3 | Y | N | PZ165876 | 34.388.196 | 47.707.334 |
| CyprMar1 | Iran | Jarahi | *Cyprinion macrostomum* | CyprMar1 | Y | Y | PZ165877 | 30.868.795 | 5.016.194 |
| CyprMar3 | Iran | Jarahi | *Cyprinion macrostomum* | CyprMar3 | Y | Y | PZ165878 | 30.868.795 | 5.016.194 |
| CyprMar4 | Iran | Jarahi | *Cyprinion macrostomum* | CyprMar4 | Y | N | PZ165879 | 30.868.795 | 5.016.194 |
| CyprMar6 | Iran | Jarahi | *Cyprinion macrostomum* | CyprMar6 | Y | N | PZ165880 | 30.868.795 | 5.016.194 |
| Ex54B1 | Iran | Tigris | *Cyprinion macrostomum* | FSJF-2197 | Y | N | PZ165881 | 30.737.556 | 51.497.833 |
| Ex54B2 | Iran | Tigris | *Cyprinion macrostomum* | FSJF-2197 | Y | N | PZ165882 | 30.737.556 | 51.497.833 |
| Ex54B3 | Iran | Tigris | *Cyprinion macrostomum* | FSJF-2197 | Y | N | PZ165883 | 30.737.556 | 51.497.833 |
| Ex62H10 | Turkey | Euphrates | *Cyprinion macrostomum* | FSJF-DNA-2413 | Y | N | PZ165884 | 39.313.630 | 37.469.460 |
| Ex62H11 | Turkey | Euphrates | *Cyprinion macrostomum* | FSJF-DNA-2413 | Y | N | PZ165885 | 39.313.630 | 37.469.460 |
| Ex82D9 | Iran | Shoor | *Cyprinion macrostomum* | FSJF-DNA-149 | Y | N | PZ165886 | 28.787.583 | 54.372.017 |
| Ex82D10 | Iran | Shoor | *Cyprinion macrostomum* | FSJF-DNA-149 | Y | N | PZ165887 | 28.787.583 | 54.372.017 |
| Ex85B2 | Iran | Kajou | *Cyprinion microphthalmum* | - | Y | N | PZ165888 | 26.259.564 | 60.746.447 |
| Ex85B3 | Iran | Kajou | *Cyprinion microphthalmum* | - | Y | N | PZ165889 | 26.259.564 | 60.746.447 |
| Ex85B4 | Iran | Kajou | *Cyprinion microphthalmum* | - | Y | N | PZ165890 | 26.259.564 | 60.746.447 |
| Ex90C7 | Iran | Tigris | *Cyprinion macrostomum* | ZM-CBSU-Cy480 | Y | N | PZ165891 | 33.046.111 | 46.575.556 |
| Ex90C8 | Iran | Zohreh | *Cyprinion macrostomum* | ZM-CBSU-Cy423 | Y | N | PZ165892 | 31.185.111 | 51.525.917 |
| Ex90F6 | Iran | Tigris | *Cyprinion macrostomum* | ZM-CBSU-Cy432 | Y | N | PZ165893 | 31.332.806 | 48.676.083 |
| Museum Sample ID | Country | Drainage | Species | Identifier | Data available | | GenBank accession no. | Coordinates | |
|  |  |  |  |  | mtDNA | SNP |  | lat | Long |
| Ex90F7 | Iran | Tigris | *Cyprinion macrostomum* | ZM-CBSU-Cy431 | Y | N | PZ165894 | 32.045944 | 48.299889 |
| Ex91A1 | Oman | Khawd | *Cyprinion muscatense* | NHM | Y | N | PZ165895 | 23.577133 | 58.119033 |
| Ex91A2 | Oman | Khawd | *Cyprinion muscatense* | NHM | Y | N | PZ165896 | 23.577133 | 58.119033 |
| Ex91A3 | Oman | Khabbah | *Cyprinion muscatense* | NHM | Y | N | PZ165897 | 22.916933 | 58.872167 |
| Ex92C3 | Iran | Makran | *Cyprinion microphthalmum* | ZM-CBSU HRE M1132 | Y | N | PZ165898 | - | - |
| Ex92C4 | Iran | Makran | *Cyprinion microphthalmum* | ZM-CBSU HRE M1133 | Y | N | PZ165899 | - | - |
| Ex92C7 | Iran | Jazmurian | *Cyprinion microphthalmum* | ZM-CBSU HRE M1137 | Y | N | PZ165900 | - | - |
| FSJF3393JFdiv22 | Iran | Tigris | *Cyprinion macrostomum* | 3393 | Y | N | PZ165901 | 33.785702 | 48.206477 |
| FSJF3415aJFdiv22 | Iran | Minab | *Cyprinion microphthalmum* | 3415a | Y | N | PZ165902 | 27.766546 | 57.248288 |
| FSJF3415bJFdiv22 | Iran | Minab | *Cyprinion microphthalmum* | 3415b | Y | N | PZ165903 | 27.766546 | 57.248288 |
| FSJF3415cJFdiv22 | Iran | Minab | *Cyprinion microphthalmum* | 3415c | Y | N | PZ165904 | 27.766546 | 57.248288 |
| FSJF3451aJFdiv22 | Iran | Tigris | *Cyprinion macrostomum* | 3451a | Y | N | PZ165905 | 32.822019 | 46.975304 |
| FSJF3451bJFdiv22 | Iran | Tigris | *Cyprinion macrostomum* | 3451b | Y | N | PZ165906 | 32.822019 | 46.975304 |
| GBOL655A12 | Oman | Bani-Khalid | *Cyprinion muscatense* | FSJF-DNA-2579 | Y | Y | PZ165907 | 22.59528 | 59.0869 |
| GBOL655C02 | Oman | Fanja | *Cyprinion muscatense* | FSJF-DNA-2583 | Y | Y | PZ165908 | 23.45811 | 58.10682 |
| GBOL655C03 | Oman | Fanja | *Cyprinion muscatense* | FSJF-DNA-2583 | Y | Y | PZ165909 | 23.45811 | 58.10682 |
| ZM-CBSU-Cy615 | Iran | Mond | *Cyprinion tenuiradius* | ZM-CBSU HRE Cy615 | Y | N | PZ165910 | 29.181833 | 52.691167 |
| ZM-CBSU-Cy617 | Iran | Mond | *Cyprinion tenuiradius* | ZM-CBSU HRE Cy617 | Y | N | PZ165911 | 29.181833 | 52.691167 |
| ZM-CBSU-Cy734 | Iran | Sirjan | *Cyprinion microphthalmum* | ZM-CBSU HRE Cy734 | Y | N | PZ165912 | 30.032389 | 54.331806 |
| ZM-CBSU-Cy735 | Iran | Sirjan | *Cyprinion microphthalmum* | ZM-CBSU HRE Cy735 | Y | N | PZ165913 | 30.032389 | 54.331806 |
| FSJF-DNA-1426 | Türkiye | Tigris | *Cyprinion kais* | Ex55B8 | Y | Y | PZ019778 | 37.83856 | 40.69367 |
| FSJF-DNA-1426 | Türkiye | Tigris | *Cyprinion kais* | Ex55B9 | Y | Y | PZ019779 | 37.83856 | 40.693670 |
| FSJF-DNA-2273 | Iraq | Tigris | *Cyprinion kais* | Ex59D2 | Y | Y | PZ019780 | 35.959167 | 45.387500 |
| FSJF-DNA-920 | Türkiye | Tigris | *Cyprinion macrostomum* | Ex50E9 | Y | Y | PZ019788 | 37.887167 | 40.229800 |
| FSJF-DNA-920 | Türkiye | Tigris | *Cyprinion macrostomum* | Ex50E10 | Y | Y | PZ019787 | 37.887167 | 40.229800 |
| Museum Sample ID | Country | Drainage | Species | Identifier | Data available | | GenBank accession no. | Coordinates | |
|  |  |  |  |  | mtDNA | SNP |  | Lat | long |
| FSJF-DNA-2233 | Iraq | Tigris | *Cyprinion macrostomum* | Ex59A10 | Y | Y | PZ019771 | 35.808889 | 45.022222 |
| FSJF-DNA-2000 | Iran | Karvander | *Cyprinion microphthalmum* | Ex82B10 | Y | Y | PZ019786 | 27.855 | 60.767500 |
| FSJF-DNA-2000 | Iran | Karvander | *Cyprinion microphthalmum* | Ex82B9 | Y | Y | PZ019772 | 27.855 | 60.767500 |
| FSJF-DNA-2000 | Iran | Karvander | *Cyprinion microphthalmum* | Ex82B11 | Y | Y | PZ019789 | 27.855 | 60.767500 |
| FSJF-DNA-2466 | Iran | Kajou | *Cyprinion microphthalmum* | Ex85B1 | Y | Y | PZ019776 | 26.259564 | 60.746447 |
| FSJF-DNA-2585 | Oman | Bani-harus | *Cyprinion muscatense* | GBOL655C11 | Y | Y | PZ019784 | 23.18016 | 57.64741 |
| FSJF-DNA-2585 | Oman | Bani-harus | *Cyprinion muscatense* | GBOL655C12 | Y | Y | PZ019785 | 23.18016 | 57.64741 |
| NHKSA034 | SA | Khaybar | *Cyprinion acinaces* | divmxext15 | Y | Y | PZ019789 | 25.747262 | 39.260362 |
| NHKSA035 | SA | Khaybar | *Cyprinion acinaces* | divmxext16 | Y | Y | PZ019776 | 25.747262 | 39.260362 |
| NHKSA039 | SA | Khaybar | *Cyprinion acinaces* | divmxext17 | Y | Y | PZ019784 | 25.747262 | 39.260362 |
| NHKSA040 | SA | Khaybar | *Cyprinion acinaces* | WCSE2 | Y | Y | PZ019785 | 25.747262 | 39.260362 |
| NHKSA050 | SA | div-Wadis | *Cyprinion mhalense* | WCSD10 | Y | Y | PZ019789 | 21.321659 | 40.454970 |
| NHKSA051 | SA | div-Wadis | *Cyprinion mhalense* | WCSD11 | Y | Y | PZ019776 | 20.762300 | 41.231388 |
| NHKSA052 | SA | div-Wadis | *Cyprinion mhalense* | WCSD12 | Y | Y | PZ019784 | 20.762300 | 41.231388 |
