# Supplementary material for: Mito‐Nuclear Discordance and Species Boundaries in the Freshwater Fish Genus Cyprinion Revealed by Genome‐Wide SNPs
Source: Ecol Evol. 2026 Jul 31;16(8):e73893. doi: 10.1002/ece3.73893 (PMC13426011; doi:10.1002/ece3.73893)
Supplement: Supplementary file 2 — Table SII: Pairwise F ST values among Cyprinion species calculated from 14,492 SNPs. F ST values are shown below the diagonal, and associated p‐values are shown above the diagonal. [file ECE3-16-e73893-s001.doc]

Supplementary Table II Pairwise *F_ST_* values among *Cyprinion* species calculated from 14,492 SNPs. *F_ST_* values are shown below the diagonal, and associated *P-values* are shown above the diagonal.

|  | *C. kais* | *C. macrostomum* | *C. microphthalmum* | *C. muscatense* | *C. acinaces* | *C. mhalense* |
| --- | --- | --- | --- | --- | --- | --- |
| *C. kais* | 0.000 | <0.001 | <0.001 | <0.001 | <0.001 | <0.001 |
| *C. macrostomum* | 0.316 | 0.000 | <0.001 | <0.001 | <0.001 | <0.001 |
| *C. microphthalmum* | 0.511 | 0.452 | 0.000 | <0.001 | <0.001 | <0.001 |
| *C. muscatense* | 0.735 | 0.551 | 0.626 | 0.000 | <0.001 | <0.001 |
| *C. acinaces* | 0.668 | 0.456 | 0.614 | 0.907 | 0.000 | <0.001 |
| *C. mhalense* | 0.625 | 0.432 | 0.572 | 0.904 | 0.798 | 0.000 |
