# Supplementary material for: Mito‐Nuclear Discordance and Species Boundaries in the Freshwater Fish Genus Cyprinion Revealed by Genome‐Wide SNPs
Source: Ecol Evol. 2026 Jul 31;16(8):e73893. doi: 10.1002/ece3.73893 (PMC13426011; doi:10.1002/ece3.73893)
Supplement: Supplementary file 3 — Table SIII: Chronological order of currently recognized species of Cyprinion, including original combinations, authorship, and type localities. Species originally described under other genera (e.g., Scaphiodon and Barbus) are indicated by their original combinations. [file ECE3-16-e73893-s003.doc]

**Supplementary Table III** Chronological order of currently recognised species of Cyprinion, including original combinations, authorship, and type localities. Species originally described under other genera (e.g., *Scaphiodon* and *Barbus*) are indicated by their original combinations.

| **Year** | **Current species** | **Original combination** | **Authorship** | **Type locality** |
| --- | --- | --- | --- | --- |
| 1843 | *Cyprinion macrostomum* | *Cyprinion macrostomus* | Heckel, 1843 | Qweiq River, Aleppo, Syria and Tirgris River, Mosul, Iraq |
| 1843 | *Cyprinion kais* | *Cyprinion kais* | Heckel, 1843 | Qweiq River, Aleppo, Syria |
| 1847 | *Cyprinion tenuiradius* | *Cyprinion tenuiradius* | Heckel, 1847 | Kara-Agaj Region, and Lake Neiriz Basin, southern Iran |
| 1872 | *Cyprinion watsoni* | *Scaphiodon watsoni* | Day, 1872 | Indus River Basin, Pakistan |
| 1880 | *Cyprinion microphthalmum* | *Barbus microphthalmus* | Day, 1880 | Quetta, Pakistan |
| 1880 | *Cyprinion milesi* | *Barbus milesi* | Day, 1880 | A spring at Trál, Pakistan |
| 1888 | *Cyprinion muscatense* | *Scaphiodon muscatensis* | Boulenger, 1888 | Muscat Region, Oman |
| 1977 | *Cyprinion acinaces* | *Cyprinion acinaces* | Banister & Clarke, 1977 | Wadi Hadramawt Drainage, Yemen |
| 1983 | *Cyprinion mhalense* | *Cyprinion mhalense* | Alkahem & Behnke, 1983 | Wadi Mhal, southwestern Saudi Arabia |

**Refrences**

Alkahem, H. F. and Behnke, R.J. (1983) Freshwater fishes of Saudi Arabia. Fauna of Saudi Arabia 5:545-567.

Heckel, J. J.  (1843) Ichthyologie [von Syrien]. In: J. von Russegger. Reisen in Europa, Asien und Afrika, mit besonderer Rücksicht auf die naturwissenschaftlichen Verhältnisse der betreffenden Länder unternommen in den Jahren 1835 bis 1841, etc. E. Schweizerbart'sche Verlagshandlung. Stuttgart. Ichthyologie [von Syrien]. In Russegger v. 1 (pt 2): 991-1099.

 Alkahem, H. F. and Behnke, R.J. (1983) Freshwater fishes of Saudi Arabia. Fauna of Saudi Arabia 5:545-567.

Banister, K.E. and Clarke, M.A. (1977) The freshwater fishes of the Arabian Peninsula.

Boulenger, G.A. (1887) November. An account of the fishes obtained by Surgeon‐Major ASG Jayakar at Muscat, east coast of Arabia. In *Proceedings of the Zoological Society of London* (Vol. 55, No. 4, pp. 653-667). Oxford, UK: Blackwell Publishing Ltd. <https://doi.org/10.1111/j.1469-7998.1887.tb08159.x>

Banarescu, P.M. and Herzig-Straschil, B. (1995) A revision of the species of the Cyprinion macrostomus-group (Pisces: Cyprinidae). *Annalen des Naturhistorischen Museums in Wien. Serie B für Botanik und Zoologie*, pp.411-420.

Jouladeh-Roudbar, A., Vatandoust, S., Eagderi, S., Jafari-Kenari, S., & Mousavi-Sabet, H. (2015) Freshwater fishes of Iran; an updated checklist. *Aquaculture, Aquarium, Conservation & Legislation*, *8*(6), 855-909.
